# MiR‐194 regulates nasopharyngeal carcinoma progression by modulating MAP3K3 expression

**DOI:** 10.1002/2211-5463.12545

**Published:** 2018-11-26

**Authors:** Wei Yin, Lei Shi, Yanjiao Mao

**Affiliations:** ^1^ Hangzhou Cancer Hospital China; ^2^ Department of Otolaryngology‐Head and Neck Surgery Shandong Provincial Hospital Affiliated to Shandong University Jinan China

**Keywords:** MAP3K3, microRNA, miR‐194, nasopharyngeal carcinoma, tumor progression

## Abstract

Despite the recent development of treatment strategies for nasopharyngeal carcinoma, the effective management of this disease remains a challenging clinical problem. A better understanding of the regulatory roles of miR‐194 and mitogen‐activated protein kinase kinase kinase 3 (MAP3K3) in the nasopharyngeal‐carcinoma‐related gene network is required to address this issue. Here, we measured relative expression of miR‐194 in human nasopharyngeal carcinoma tissues and normal epithelial tissues by quantitative real time PCR. We transfected cultured CNE‐1 and C666‐1 cells with miR‐194 mimics, and then examined the effects on cell proliferation, cell migration and invasion. Luciferase reporter assay was used to validate the putative binding between miR‐194 and MAP3K3. We then examined the effect of knockdown and overexpression of MAP3K3 on cell tumorigenesis. Expression of miR‐194 is significantly down‐regulated in nasopharyngeal carcinoma specimens and tumor cell lines when compared with normal controls. In addition, miR‐194 suppressed tumor cell proliferation and viability, as well as migration and invasion of carcinoma cells. We found that miR‐194 binds the 3′ untranslated region of MAP3K3, and knockdown of miR‐194 inhibited nasopharyngeal carcinoma cell proliferation, migration and invasion. In accordance, overexpression of MAP3K3 reversed the inhibitory effects of miR‐194 in carcinoma cells. This study suggests that expression of miR‐194 is down‐regulated in nasopharyngeal carcinoma, and that miR‐194 can directly target MAP3K3 to regulate tumor progression. Given the pivotal involvement of MAP3K3 in nasopharyngeal carcinoma development, targeting miR‐194 may be a novel strategy for the treatment of nasopharyngeal carcinoma.

**Abbreviations**
EBV: Epstein–Barr virus
MAP3K3: mitogen‐activated protein kinase kinase kinase 3
miRNA: microRNA
MTT: 3‐(4,5‐dimethylthiazol‐2‐yl)‐2,5‐diphenyltetrazolium bromide
qRT‐PCR: quantitative real time PCR
shRNA: short hairpin RNA
UTR: untranslated region

Nasopharyngeal carcinoma is a disease in which malignant cells form in the nasopharynx tissues. It is a rare type of head and neck cancer that is particularly common in southern China and Southeast Asia 1. Distinct from most other head and neck cancers, which are predominantly treated by surgery, radiotherapy is the main treatment strategy for nasopharyngeal carcinoma 2. Owing to the application of magnetic resonance imaging, intensity‐modulated radiotherapy, as well as comprehensive chemotherapy strategies, the local control of nasopharyngeal carcinoma has been greatly improved, while the 5‐year survival rate has increased from 50% in the 1980s to 80% currently 3. However, due to the high rates of lethal conditions, low life quality after treatment, and the progressive recurrence of nasopharyngeal carcinoma, the effective management of this disease remains a challenging clinical problem 4. Better understanding of the mechanism of action underlying the progression of the nasopharyngeal carcinoma will help ameliorate the development of the treatment strategies, providing more comprehensive medical evidence for ensuring the effectiveness of the treatments.

Recent studies have linked microRNAs (miRNAs), which are 20‐ to 22‐nucleotide non‐coding RNAs that regulate the expression of their cognate target genes by specifically binding and cleaving mRNAs, to the development of cancer metastasis 5. For instance, it was shown that miR‐194 prevents liver cancer cell metastasis by targeting the 3′ untranslated region (UTR) of several genes that are involved in cancer metastasis and epithelial–mesenchymal transition 6. Another study showed that miR‐192 and miR‐215 were significantly reduced in a variety of colon cancer samples, acting as effectors of the tumor suppressor *p53* gene 7, whereas p53‐responsive miR‐194 inhibits tumor by binding with the 3′ UTR of the *THBS1* gene, which encodes an endogenous inhibitor of angiogenesis, thrombospondin‐1, and thus promotes angiogenesis in colon cancer 8. miR‐194 expression is also found to be significantly negatively associated with metastasis in clinical specimens of non‐small cell lung cancer 9. In addition, miR‐194 has been suggested to be a putative tumor suppressor in multiple myeloma and gastric cancer 10, 11, 12. However, its function in nasopharyngeal carcinoma is still unclear.

The mitogen‐activated protein kinase kinase kinase 3 (MAP3K3) functions as an upstream regulator of the mitogen‐activated protein kinase signaling pathway, modulating various biological functions including cell proliferation, differentiation, migration and apoptosis 13. Increased expression of MAP3K3 in ovarian cancer, esophageal and breast cancer has been reported to be associated with tumorigenesis 14.

Although aberrant expression of miR‐194 and MAP3K3 in different cancer cells has been shown to suppress tumor cell invasion and metastasis, little is known about their role in regulating the progression of nasopharyngeal carcinoma. The present study was designed to investigate whether miR‐194 and MAP3K3 are involved and correlated in the nasopharyngeal‐carcinoma‐related gene network.

## Materials and methods

### Clinical specimens and cell lines

Human nasopharyngeal carcinoma tissues (*n* = 15) and normal nasopharyngeal epithelial tissues (*n* = 39) were obtained from patients diagnosed at hospital. The protocol for the use of human tissues was approved by the Institutional Research Ethics Committee in Hospital, and patients’ consent was obtained prior to the surgical resection. All specimens were snap‐frozen in liquid nitrogen and stored at −80 °C for subsequent use. Human nasopharyngeal epithelial cell line NP69 and nasopharyngeal carcinoma cell lines CNE‐1, CNE‐2, HONE‐1, HNE‐1, C666‐1 and SUNE‐1 were purchased from American Type Culture Collection (Manassas, VA, USA) and cultured in RPMI‐1640 supplemented with 5% FBS at 37 °C in an atmosphere of 5% CO_2_. The experiments were undertaken with the informed written consent of each subject. The study methodologies conformed to the standards set by the Declaration of Helsinki.

### miRNA extraction and quantitative real time PCR

According to the manufacturer's instruction, total miRNA from the patients’ tissues and cultured cells was extracted using the mirVana miRNA Isolation Kit (Thermo Fisher Scientific, Waltham, MA, USA). cDNA was synthesized using the Taqman miRNA reverse transcription kit (Thermo Fisher Scientific), and expression levels of miR‐194 were quantified using TaqMan hsa‐miR‐194 assay (Thermo Fisher Scientific). Quantitative real time PCR (qRT‐PCR) was performed using the CFX‐1000 Real‐time PCR system (Bio‐Rad, Hercules, CA, USA).

### Cell proliferation assay

To determine the cell growth in the presence of miRNA mimics, CNE‐1 and C666 cells were transfected with 20 pmol of miR‐Ctrl and miR‐194 mimics (Genepharma, Shanghai, China) using the Lipofectamine RNAiMAX transfection reagent (Thermo Fisher Scientific) following the manufacturer's protocol and cultured for 24 h. Further, CNE‐1 and C666 cells were seeded at 1 × 10^5^ cells per well into a six‐well plate and cultured for 72 h, followed by digestion, and the total cell number of each well was counted three times using the Z1 particle counter (Beckman Coulter, Inc., Brea, CA, USA). For the cell viability assay, miRNA‐treated CNE‐1 and C666 cells were seeded at 1 × 10^3^ cells per well into a 96‐well plate. After 72 h incubation, cells were detected by 3‐(4,5‐dimethylthiazol‐2‐yl)‐2,5‐diphenyltetrazolium bromide (MTT) assay. The plates were read at 570 nm on an automated microplate reader (Molecular Devices, Sunnyvale CA, USA).

### Cell migration and invasion assays

A cell migration assay was performed using Transwell (Corning Inc., Corning, NY, USA). A cell invasion assay was performed using the same Transwell chamber coated with Matrigel (BD Biosciences, San Jose, CA, USA). Briefly, 1 × 10^5^ CNE‐1 and C666 cells in serum‐free DMEM were added to the upper chamber of the transwell insert and DMEM with 10% FBS was added to the lower wells of the chambers. Cells were allowed to migrate at 37 °C for 12 h. Cells that migrated through the membrane to the lower surface of the transwell were fixed with methanol and stained with crystal violet for 20 min. The migrated cells attached to the underside of the inserts were photographed and counted under a light microscope (Nikon, Chiyoda, Japan).

### Luciferase assays

The putative target sites of miR‐194 on the 3′ UTR of MAP3K3 were predicted at http://www.targetscan.org/vert_71. A total of 3 × 10^4^ CNE‐1 and C666 cells were seeded into 96‐well plates. Twenty‐four hours after plating, the cells were transfected with a psiCHECK2 vector encoding the wild‐type and mutated 3′ UTR of MAP3K3. After incubation for 48 h, the cells were lysed in 1× Passive Lysis Buffer and assayed with the Dual‐Luciferase^®^ Reporter Assay System (Promega, Madison, WI, USA) to measure the luciferase activity as described in the instructions.

### Western blot

A total of 20 μg of protein from CNE‐1 and C666 cell lysates was separated by 10% SDS/PAGE and transferred to poly(vinylidene difluoride) membrane (Bio‐Rad). The membranes were blocked with 5% non‐fat milk in Tris‐buffered saline with 0.1% Tween 20 (TBST) for 2 h and subsequently incubated with primary antibody against MAP3K3 and β‐actin (Cell Signaling Technology, Danvers, MA, USA) overnight at 4 °C. Membranes were washed three times with TBST and incubated with secondary antibodies for 1 h. All membranes were detected using enhanced chemiluminescence (Thermo Fisher Scientific), and protein expression was analyzed using image‐pro plus software 6.0 (Media Cybernetics, Rockville, MD, USA).

### Stable knockdown and overexpression of MAP3K3 in nasopharyngeal carcinoma cells

For knockdown study, short hairpin RNAs (shRNAs) targeting MAP3K3 (shMAP3K3 #1 and shMAP3K3 #2) and non‐target control (shCtrl) were purchased from Shanghai Genepharma Inc. and delivered to CNE‐1 and C666 cells. Stably transduced cells were selected by puromycin at 1 mg·mL^−1^ and subjected to cell number assay, MTT assay, and transwell migration and invasion assay as described earlier.

For the overexpression study, MAP3K3 expression vector and control vector were purchased from Shanghai Genepharma Inc., and transfected into CNE‐1 and C666 cells using Lipofectamine 2000 (Thermo Fisher Scientific). Cells with stable overexpression were selected using 1 mg·mL^−1^ of G418, and further transfected miR‐194 mimic and miR ‐Ctrl, followed by the MAP3K3 protein expression, cell growth, cell migration and invasion assays.

### Statistical analysis

All statistical analyses were carried out using the spss 11.0 statistical software package (SPSS Inc., Chicago, IL, USA). Statistical comparison of the two groups was conducted using Student's *t* test. Comparisons between multiple groups were made using one‐ or two‐way ANOVA. All data represent mean ± standard deviation (SD) from at least three independent experiments. A *P*‐value smaller than 0.05 was considered statistically significant.

## Results

### miR‐194 is down‐regulated in nasopharyngeal carcinoma tissues and cell lines

**Figure 1:**
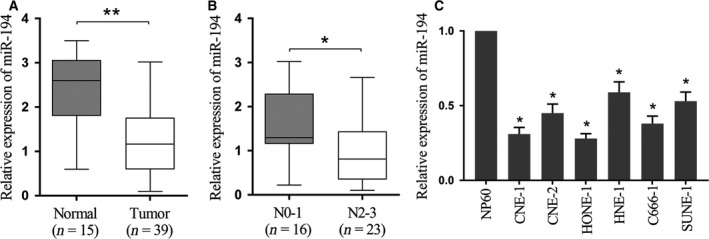
miR‐194 is down‐regulated in nasopharyngeal carcinoma tissues and cell lines. (A) Relative expression of miR‐194 in normal nasopharyngeal epithelial tissue (*n* = 15) and nasopharyngeal carcinoma (*n* = 39) tissues was detected by qRT‐PCR. **P < 0.01, Student's *t* test. (B) Relative expression of miR‐194 in nasopharyngeal epithelial tissues with low (N0–1, *n* = 16) or high (N2–3, *n* = 23) level regional lymph node metastasis was detected by qRT‐PCR. *P < 0.05, Student's *t* test. (C) Relative expression of miR‐194 in nasopharyngeal epithelial cell line NP69 and nasopharyngeal carcinoma cell lines (CNE‐1, CNE‐2, HONE‐1, HNE‐1, C666‐1 and SUNE‐1). U6 was used as an endogenous control. *P < 0.05, one‐way ANOVA.

We first detected the relative expression of miR‐194 in 15 normal nasopharyngeal epithelial tissues and 39 human nasopharyngeal carcinoma specimens, out of which there were 16 samples with low levels of regional lymph node metastasis, and 23 samples with high levels of regional lymph node metastasis. The result showed that the expression of miR‐194 in nasopharyngeal carcinoma tissues was significantly down‐regulated (*P* < 0.01, Fig. 1A), while the expression level was lower in the samples with high regional lymph node metastasis than in samples with low regional lymph node metastasis (*P* < 0.05, Fig. 1B). Moreover, expression levels of miR‐194 in different nasopharyngeal carcinoma cells, including CNE‐1, CNE‐2, HONE‐1, HNE‐1, C666‐1 and SUNE‐1, were significantly lower than in the cultured human nasopharyngeal epithelial NP69 cells (Fig. 1C). These observations highlighted the clinical relevance of miR‐194 in nasopharyngeal carcinoma.

### miR‐194 suppresses nasopharyngeal carcinoma cell proliferation, migration and invasion

**Figure 2:**
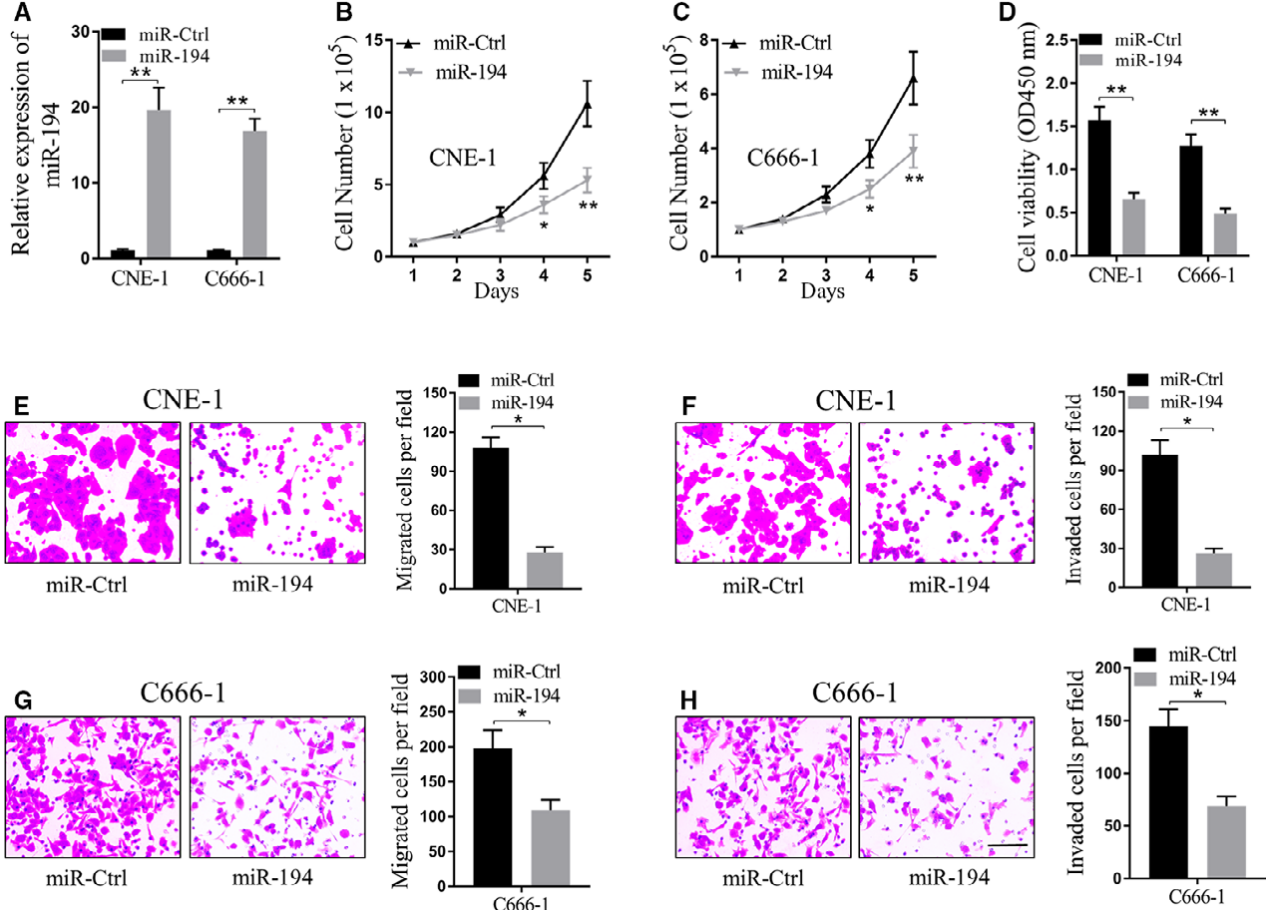
miR‐194 suppresses nasopharyngeal carcinoma cell proliferation, migration and invasion. (A) Relative miR‐194 expression after transfection with miR‐Ctrl and miR‐194 mimics. miR‐194 levels were measured via qRT‐PCR and normalized to the level of U6 in CNE‐1 and C666‐1 cells. **P < 0.01, Student's *t* test. (B,C) CNE‐1 (B) and C666‐1 (C) cells were subjected to cell number assay every 24 h. *P < 0.05, **P < 0.01, two‐way ANOVA. (D) CNE‐1 and C666‐1 cells subjected to MTT assay after plating for 72 h. **P < 0.01, Student's *t* test. (E,G) CNE‐1 (E) and C666‐1 (G) cells were subjected to transwell migration assay. *P < 0.05, Student's *t* test. (F,H) CNE‐1 (F) and C666‐1 (H) cells were subjected to transwell invasion assay. *P < 0.05, Student's *t* test. Scale bar, 50 μm. Data are mean ± SD of three independent experiments, each was measured in triplicate.

In order to investigate the role of miR‐194 in the progression of nasopharyngeal carcinoma, miR‐194 mimics were transfected into nasopharyngeal carcinoma cell lines CNE‐1 and C666‐1. Firstly qRT‐PCR results showed that mir‐194 levels were steadily high in both CNE‐1 and C666‐1 cells (*P* < 0.01, Fig. 2A). The cell number assay for five consecutive days showed that both CNE‐1 and C666‐1 cells transfected with miR‐194 grew more slowly than cells transfected with miR‐Ctrl (Fig. 2B,C), and the cell viability of the cells with miR‐194 transfection was significantly lower than the control group (*P* < 0.01, Fig. 2D). In line with this observation, we showed that both the cell migration and the cell invasion were significantly impaired by miR‐194 transfection when compared with cells transfected with miR‐Ctrl (*P* < 0.05 for both, Fig. 2E–H), suggesting the potential inhibitory function of miR‐194 in cancer progression.

### MAP3K3 is a direct target of miR‐194

**Figure 3:**
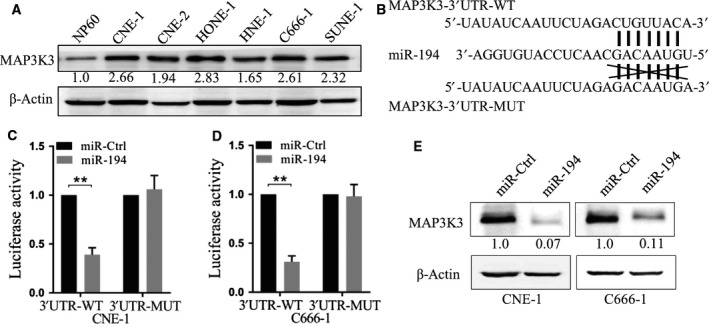
MAP3K3 is a direct target of miR‐194. (A) Relative expression of MAP3K3 in nasopharyngeal epithelial cell line NP69 and nasopharyngeal carcinoma cell lines (CNE‐1, CNE‐2, HONE‐1, HNE‐1, C666‐1 and SUNE‐1). β‐Actin was used as an endogenous control. (B) Sequences of miRNA and the potential miRNA binding sites at the 3′ UTR of MAP3K3. (C, D) Relative luciferase activity of CNE‐1 and C666‐1 cells after co‐transfection with wild‐type or mutant MAP3K3 3′ UTR reporter genes and miR‐ctrl or miR‐194 mimic. **P < 0.01, Student's *t* test. (E) miR‐194 over‐expression reduced the protein expression of MAP3K3 in CNE‐1 and C666‐1 cells. Data are mean ± SD of three independent experiments, each measured in triplicate.

Several studies have shown that MAP3K3 expression in tumor cells was relevant to cancer progression. Therefore, we investigated the possible correlation between miR‐194 and MAP3K3. MAP3K3 expression was much higher in nasopharyngeal carcinoma cell lines (CNE‐1, CNE‐2, HONE‐1, HNE‐1, C666‐1 and SUNE‐1) when compared to that in nasopharyngeal epithelial cell line NP69 (Fig. 3A). *In silico* analysis revealed that the 3′ UTR of MAP3K3 contains the putative binding site of miR‐194 (Fig. 3B). To confirm the binding between miR‐194 and MAP3K3 3′ UTR, the luciferase reporter assay was performed using the WT or mutated MAP3K3 3′ UTR‐coupled luciferase reporter. We found that ectopic expression of miR‐194 significantly decreased the luciferase signal of WT MAP3K3 3′ UTR in both CNE‐1 and C666‐1 cells, in comparison with the miR‐Ctrl. These suppressive effects were abolished with a mutated miR‐194 binding site of MAP3K3 (*P* < 0.01, Fig. 3C,D). Furthermore, western blot results indicated that miR‐194 overexpression significantly reduced the protein expression of endogenous MAP3K3 in CNE‐1 and C666‐1 cells (Fig. 3E). All of these results indicated that MAP3K3 is a direct target of miR‐194, and miR‐194 inhibits the nasopharyngeal carcinoma progression through modulating MAP3K3.

### MAP3K3 knockdown suppresses nasopharyngeal carcinoma cell proliferation, migration and invasion

**Figure 4:**
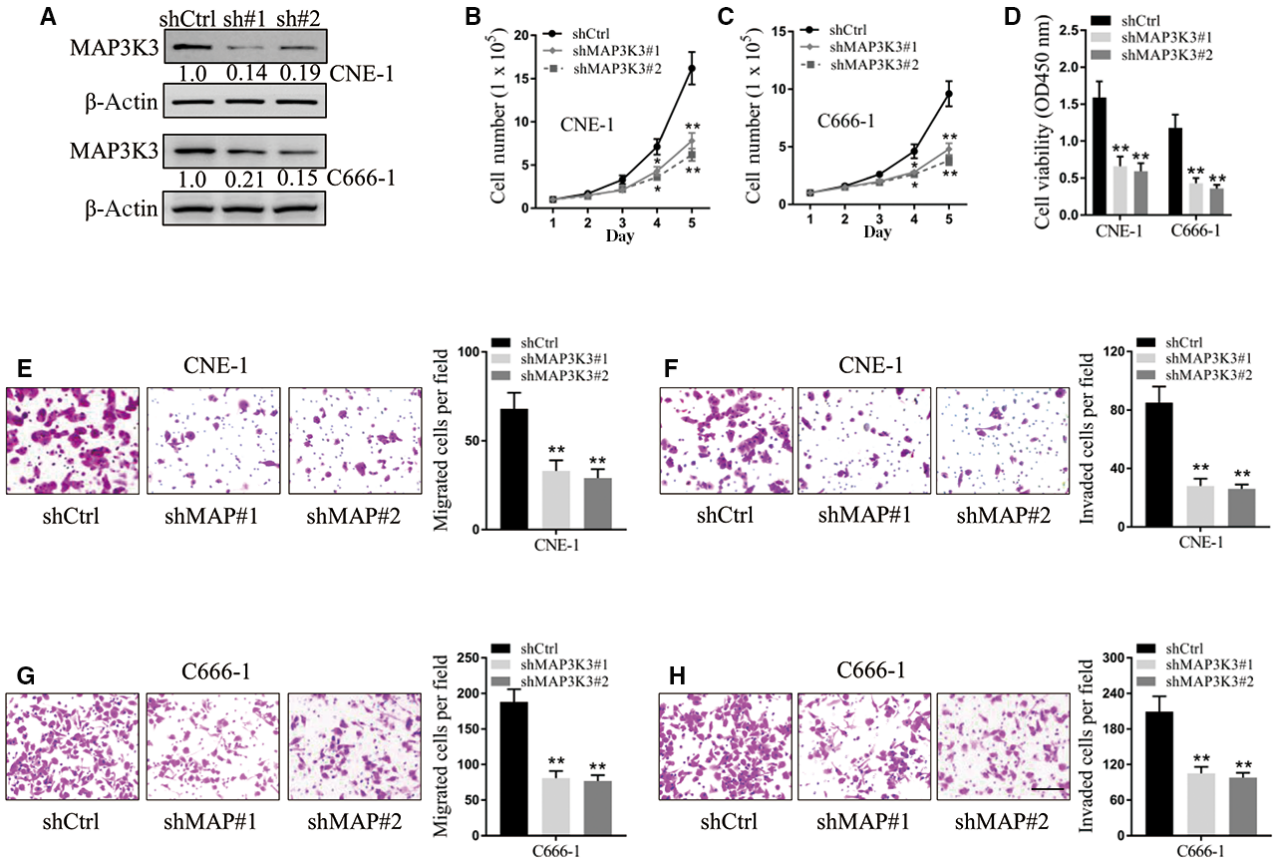
MAP3K3 knockdown suppresses nasopharyngeal carcinoma cell proliferation, migration and invasion. (A) Relative expression of MAP3K3 in CNE‐1 and C666‐1 cell lines after knockdown of MAP3K3. β‐Actin was used as an endogenous control. (B, C) CNE‐1 (B) and C666‐1 (C) cells were subjected to cell number assay every 24 h. *P < 0.05, **P < 0.01, two‐way ANOVA. (D) CNE‐1 C666‐1 cells subjected to MTT assay after plating for 72 h. **P < 0.01, one‐way ANOVA. (E, G) CNE‐1 (E) and C666‐1 (G) cells were subjected to transwell migration assay. **P < 0.01, one‐way ANOVA. (F, H) CNE‐1 (F) and C666‐1 (H) cells were subjected to transwell invasion assay. **P < 0.01, one‐way ANOVA. Scale bar, 50 μm. Data are mean ± SD of three independent experiments, each measured in triplicate.

To investigate whether MAP3K3 knockdown could interfere the proliferation of nasopharyngeal carcinoma cells *in vitro*, cell number and MTT assays were performed to evaluate CNE‐1 and C666‐1 cells transfected with shRNAs targeting MAP3K3. First, MAP3K3 silencing efficacy was verified by the reduced expression of MAP3K3 in CNE‐1 and C666‐1 cell lines after knockdown of MAP3K3 (Fig. 4A). Our results showed that the cell number assay for five consecutive days showed that both CNE‐1 and C666‐1 cells transfected with MAP3K3 shRNAs #1 and #2 grew more slowly than the cells transfected with shCtrl (Fig. 4B,C), and the cell viabilities in the cells with MAP3K3 shRNAs were significantly lower than the control group (*P* < 0.01 for all, Fig. 4D). Additionally, the cell migration and invasion assays showed that both CNE‐1 and C666‐1 cells transfected with MAP3K3 shRNAs #1 and #2 had significantly impaired cell migrated and invaded cells (*P* < 0.01 for all, Fig. 4E–H).

### Overexpression of MAP3K3 rescued the inhibition of miR‐194 in nasopharyngeal carcinoma cells

**Figure 5:**
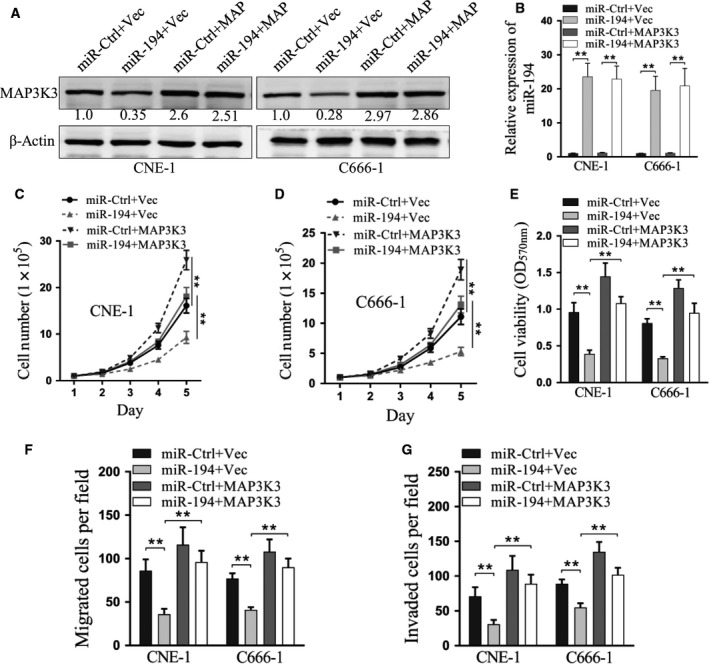
Overexpression of MAP3K3 rescued the inhibition caused by miR‐194 in nasopharyngeal carcinoma cells. (A) CNE‐1 and C666‐1 cells were transfected with miR‐Ctrl or miR‐194 mimics in MAP3K3‐overexpressing cells, and MAP3K3 protein expression was analyzed by western blot. (B) Relative miR‐194 expression after transfection with miR‐Ctrl and miR‐194 mimics. miR‐194 levels were measured via qRT‐PCR and normalized to the level of U6 in CNE‐1 and C666‐1 cells. **P < 0.01, one‐way ANOVA. (C–E) Effects of restoration of MAP3K3 on cell growth were detected by cell number assay (C, D) (**P < 0.01, two‐way ANOVA) and MTT assay (E) (**P < 0.01, one‐way ANOVA). (F, G) Effects of restoration of MAP3K3 on migration and invasion determined by transwell migration (F) and invasion (G) assays. **P < 0.01, one‐way ANOVA. Data are mean ± SD of three independent experiments, each measured in triplicate.

To investigate whether MAP3K3 overexpression could impact the inhibition of miR‐194 on nasopharyngeal carcinoma cells *in vitro*, CNE‐1 and C666‐1 cells with or without stable MAP3K3 overexpression were transfected with miR‐194. Consistent with previously described results, cells with miR‐194 demonstrated lower MAP3K3 expression, but the expression levels of MAP3K3 in the MAP3K3 overexpression cells were not influenced by miR‐194 (Fig. 5A). miR‐194 expression after transfection with miR‐194 mimics was much higher than with transfection with miR‐Ctrl (Fig. 5B). However, the cell number of both CNE‐1 and C666‐1 cells transfected with miR‐194 were significantly lower than the cells transfected with miR‐Ctrl in both normal and MAP3K3‐overexpressing cells (Fig. 5C,D), and the cell viabilities of the cells transfected with miR‐Ctrl of both normal and MAP3K3‐overexpressing cells were lower than the control group (*P* < 0.01 for all, Fig. 5E). Additionally, the cell migration and invasion assays showed that both CNE‐1 and C666‐1 cells transfected with miR‐194 had significantly down‐regulated cell migration and invasion compared with cells transfected with miR‐Ctrl (*P* < 0.01, respectively, Fig. 5F,G).

## Discussion

Nasopharyngeal carcinoma is a serious health problem in some demographic regions around the world. A great number of clinical studies have shown that the expression of miRNAs are dynamically regulated in the carcinomatosis, and miRNAs could be used as potential molecular biomarkers for human malignancies. In the current study, we showed that miR‐194 is critically involved in nasopharyngeal carcinoma progression. The miR‐194 is significantly down‐regulated in nasopharyngeal carcinoma specimens compared with normal nasopharyngeal epithelial tissues. Similar results were also observed in different nasopharyngeal carcinoma cell lines. MiR‐194 suppressed malignant phenotypes of nasopharyngeal carcinoma including tumor cell proliferation and viability, as well as migration and invasion of carcinoma cells. Computational *in silico* analysis uncovered that miR‐194 putatively binds the 3′ UTR of MAP3K3, and luciferase reporter assay confirmed MAP3K3 as a direct target of miR‐194. In addition, we found MAP3K3 knockdown inhibited nasopharyngeal carcinoma cell proliferation, migration and invasion. In accordance, overexpressing MAP3K3 reversed the inhibition effects of miR‐194 in the tested carcinoma cells, consolidating the direct influence of miR‐194 on MAP3K3.

Among all the reported studies, there are a number addressing the clinical impact of miRNAs in nasopharyngeal carcinoma on small or large scales. Microarray‐based serum miRNA profiling revealed that four miRNAs including miR‐17, miR‐20a, miR‐29c, and miR‐223 were expressed differently in the serum of nasopharyngeal carcinoma patients compared with non‐cancerous controls 15. Regarding the studies on tumor tissues, a study on miRNA expression profiling in eight NPC tissues and four normal nasopharyngeal tissues identified 34 differently expressed miRNAs in two categories 16. In our study, in addition to the comparison of the expression profile of miR‐194 in the carcinoma and normal tissues, differential expression among the carcinoma at different clinical stages, which was assessed on the basis of the regional lymph node metastasis classification system, was also explored. Identification of miRNA expression in patients with different lymph node metastasis levels would be helpful to facilitate the understanding of the mechanisms underlying the progression of nasopharyngeal carcinoma.

Due to the infrequency of nasopharyngeal carcinoma in most part of the world and the limited availability of clinical specimens, nasopharyngeal carcinoma cell lines have been established as important models to study its pathophysiology 17. One of the intriguing features of this epithelial carcinoma is its constant association with Epstein–Barr virus (EBV) infection. Studies have shown that EBV plays a key role in the pathogenesis of the tumor phenotype in nasopharyngeal carcinoma 18. The majority of nasopharyngeal carcinoma cell lines were established around a few decades ago, and only some of them, such as C666‐1 used in the current study, stably harbor the EBV genome and are relatively poorly differentiated 19. According to the published reports, CNE‐1 was originally established from well‐differentiated squamous cells 20. In other word, we investigated the effects of miR‐194 in the well‐differentiated nasopharyngeal carcinoma cell line CNE‐1 and the poorly differentiated nasopharyngeal carcinoma cell lines C666‐1, the results strongly suggesting that miR‐194 is actively involved in the progression of nasopharyngeal carcinoma at differentiation states.

MAP3K3 is widely expressed in various tissues and is activated by numerous extracellular stimuli to regulate biological processes including cell proliferation and differentiation 21. Recent studies have identified potentially clinical roles of MAP3K3 as an oncogene that promotes cancer progression in several different malignancies including non‐small‐cell lung cancer, ovarian carcinoma, lung cancer and renal clear cell carcinoma 14, 21, 22, 23. However, the clinical significance of MAP3K3 expression in nasopharyngeal carcinoma remains elusive. In this study, we confirmed the knockdown of MAP3K3 in human nasopharyngeal carcinoma cell lines by shRNA significantly inhibited the nasopharyngeal carcinoma cell proliferation, migration and invasion, indicating the prognostic role of MAP3K3 in the pathogenesis of nasopharyngeal carcinoma. Although we explored the role of miR‐194 on MAP3K3, it would be interesting to investigate the expression changes of MAP3K3 in tumor tissue specimens to decipher its independent tumorigenesis in nasopharyngeal carcinoma.

There are more than 1900 human miRNAs that have been documented in different miRBase databases, making miRNA one of the largest gene families in eukaryotes 24. Each of miRNAs could bind to various locations of hundreds of transcripts 25. In terms of miR‐194, the function of miR‐194 in different cancers is often associated with varying targets. A previous study showed that miR‐194 can target bone morphogenetic protein 1 (BMP1) and kip1, leading to suppression of transforming growth factor β activity and down‐regulation of the expression of key oncogenic genes such as matrix metalloproteinases MMP2 and MMP9, and thus suppress the metastasis of non‐small cell lung cancer 9. MiR‐194 inhibits gastric cancer progression by down‐regulating transcription factor forkhead box protein M1 (FoxM1) 26. MiR‐194 can also promote the tumorigenesis of ovarian carcinoma by directly targeting protein tyrosine phosphatase nonreceptor type 12 (PTPN12) 27. In pancreatic ductal adenocarcinoma cells, miR‐194 overexpression increased tumor growth and invasion through the suppression of dachsund homolog 1 (DACH1) 28. Overall, the roles of miR‐194 in tumor cells are not in agreement and seem controversial. Using computational analysis, we found miR‐194 binds to the 3′ UTR of MAP3K3, while the knockdown of MAP3K3 suppresses cancer cell regression. The luciferase reporter assay showed that miR‐194 did not bind to the mutated MAP3K3, indicating the specificity of the binding between these two partners. Here, we for the first time reported a function of miR‐194 in nasopharyngeal carcinoma and established an association between miR‐194 and MAP3K3, which is in line with the inhibitory role of miR‐194 in cancer regression.

In summary, the current work indicates that the miR‐194 is a potent tumor modulator in nasopharyngeal carcinoma. MiR‐194‐can directly target MAP3K3 to regulate tumor progression. As MAP3K3 is critically involved in nasopharyngeal carcinoma development, exploiting the suppressive role of miR‐194 on nasopharyngeal carcinoma might be an effective rationale for diagnosis and treatment of nasopharyngeal carcinoma.

## Conflict of interest

The authors declare no conflict of interest.

## Author contributions

WY, LS acquired, analyzed and interpreted the data. YM designed the project and wrote the paper.

## References

[1] Cao SM , Simons MJ and Qian CN (2011) The prevalence and prevention of nasopharyngeal carcinoma in China. Chin J Cancer 30, 114–119. 21272443 10.5732/cjc.010.10377 PMC4013340

[2] Lee AW , Lin JC and Ng WT (2012) Current management of nasopharyngeal cancer. Semin Radiat Oncol 22, 233–244. 22687948 10.1016/j.semradonc.2012.03.008

[3] Su SF , Han F , Zhao C , Huang Y , Chen CY , Xiao WW , Li JX and Lu TX (2011) Treatment outcomes for different subgroups of nasopharyngeal carcinoma patients treated with intensity‐modulated radiation therapy. Chin J Cancer 30, 565–573. 21801605 10.5732/cjc.010.10547 PMC4013407

[4] Xu T , Tang J , Gu M , Liu L , Wei W and Yang H (2013) Recurrent nasopharyngeal carcinoma: a clinical dilemma and challenge. Curr Oncol 20, e406–e419. 24155638 10.3747/co.20.1456 PMC3805410

[5] Bartel DP (2004) MicroRNAs: genomics, biogenesis, mechanism, and function. Cell 116, 281–297. 14744438 10.1016/s0092-8674(04)00045-5

[6] Meng Z , Fu X , Chen X , Zeng S , Tian Y , Jove R , Xu R and Huang W (2010) miR‐194 is a marker of hepatic epithelial cells and suppresses metastasis of liver cancer cells in mice. Hepatology 52, 2148–2157. 20979124 10.1002/hep.23915 PMC3076553

[7] Braun CJ , Zhang X , Savelyeva I , Wolff S , Moll UM , Schepeler T , Orntoft TF , Andersen CL and Dobbelstein M (2008) p53‐Responsive micrornas 192 and 215 are capable of inducing cell cycle arrest. Cancer Res 68, 10094–10104. 19074875 10.1158/0008-5472.CAN-08-1569 PMC2836584

[8] Sundaram P , Hultine S , Smith LM , Dews M , Fox JL , Biyashev D , Schelter JM , Huang Q , Cleary MA , Volpert OV *et al* (2011) p53‐responsive miR‐194 inhibits thrombospondin‐1 and promotes angiogenesis in colon cancers. Cancer Res 71, 7490–7501. 22028325 10.1158/0008-5472.CAN-11-1124 PMC3242824

[9] Wu X , Liu T , Fang O , Leach LJ , Hu X and Luo Z (2014) miR‐194 suppresses metastasis of non‐small cell lung cancer through regulating expression of BMP1 and p27(kip1). Oncogene 33, 1506–1514. 23584484 10.1038/onc.2013.108

[10] Pichiorri F , Suh SS , Rocci A , De Luca L , Taccioli C , Santhanam R , Zhou W , Benson DM Jr , Hofmainster C , Alder H *et al* (2010) Downregulation of p53‐inducible microRNAs 192, 194, and 215 impairs the p53/MDM2 autoregulatory loop in multiple myeloma development. Cancer Cell 18, 367–381. 20951946 10.1016/j.ccr.2010.09.005 PMC3561766

[11] Song Y , Zhao F , Wang Z , Liu Z , Chiang Y , Xu Y , Gao P and Xu H (2012) Inverse association between miR‐194 expression and tumor invasion in gastric cancer. Ann Surg Oncol 19 (Suppl. 3), S509–S517. 21845495 10.1245/s10434-011-1999-2

[12] Dong P , Kaneuchi M , Watari H , Hamada J , Sudo S , Ju J and Sakuragi N (2011) MicroRNA‐194 inhibits epithelial to mesenchymal transition of endometrial cancer cells by targeting oncogene BMI‐1. Mol Cancer 10, 99. 21851624 10.1186/1476-4598-10-99 PMC3173388

[13] Chang L and Karin M (2001) Mammalian MAP kinase signalling cascades. Nature 410, 37–40. 11242034 10.1038/35065000

[14] He Y , Wang L , Liu W , Zhong J , Bai S , Wang Z , Thomas DG , Lin J , Reddy RM , Ramnath N *et al* (2015) MAP3K3 expression in tumor cells and tumor‐infiltrating lymphocytes is correlated with favorable patient survival in lung cancer. Sci Rep 5, 11471. 26088427 10.1038/srep11471 PMC4650617

[15] Zeng X , Xiang J , Wu M , Xiong W , Tang H , Deng M , Li X , Liao Q , Su B , Luo Z *et al* (2012) Circulating miR‐17, miR‐20a, miR‐29c, and miR‐223 combined as non‐invasive biomarkers in nasopharyngeal carcinoma. PLoS ONE 7, e46367. 23056289 10.1371/journal.pone.0046367 PMC3466268

[16] Li T , Chen JX , Fu XP , Yang S , Zhang Z , Chen KhH and Li Y (2011) microRNA expression profiling of nasopharyngeal carcinoma. Oncol Rep 25, 1353–1363. 21373758 10.3892/or.2011.1204

[17] Strong MJ , Baddoo M , Nanbo A , Xu M , Puetter A and Lin Z (2014) Comprehensive high‐throughput RNA sequencing analysis reveals contamination of multiple nasopharyngeal carcinoma cell lines with HeLa cell genomes. J Virol 88, 10696–10704. 24991015 10.1128/JVI.01457-14 PMC4178894

[18] Lo KW , To KF and Huang DP (2004) Focus on nasopharyngeal carcinoma. Cancer Cell 5, 423–428. 15144950 10.1016/s1535-6108(04)00119-9

[19] Cheung ST , Huang DP , Hui AB , Lo KW , Ko CW , Tsang YS , Wong N , Whitney BM and Lee JC (1999) Nasopharyngeal carcinoma cell line (C666‐1) consistently harbouring Epstein‐Barr virus. Int J Cancer 83, 121–126. 10449618 10.1002/(sici)1097-0215(19990924)83:1<121::aid-ijc21>3.0.co;2-f

[20] Gu SY , Tang WP , Zeng Y , Zhao ML , Zhao EWP , Deng WH and Li K (1983) An epithelial cell line established from poorly differentiated nasopharyngeal carcinoma. Chin J Cancer 2, 70–72.

[21] Jia W , Dong Y , Tao L , Pang L , Ren Y , Liang W , Jiang J , Cheng G , Zhang WJ , Yuan X *et al* (2016) MAP3K3 overexpression is associated with poor survival in ovarian carcinoma. Hum Pathol 50, 162–169. 26997451 10.1016/j.humpath.2015.12.011

[22] Zhao L , Ni X , Zhao L , Zhang Y , Jin D , Yin W , Wang D and Zhang W (2018) MiroRNA‐188 acts as tumor suppressor in non‐small‐cell lung cancer by targeting MAP3K3. Mol Pharm 15, 1682–1689. 29528232 10.1021/acs.molpharmaceut.8b00071

[23] Lu H , Cao X , Chen Q , Chen L , Chen L and Gan M (2015) The expression and role of MEKK3 in renal clear cell carcinoma. Anat Rec (Hoboken) 298, 727–734. 25388155 10.1002/ar.23093

[24] Kozomara A and Griffiths‐Jones S (2011) miRBase: integrating microRNA annotation and deep‐sequencing data. Nucleic Acids Res 39, D152–D157. 21037258 10.1093/nar/gkq1027 PMC3013655

[25] Friedman RC , Farh KK , Burge CB and Bartel DP (2009) Most mammalian mRNAs are conserved targets of microRNAs. Genome Res 19, 92–105. 18955434 10.1101/gr.082701.108 PMC2612969

[26] Wang S , Jiao B , Geng S , Ma S , Liang Z and Lu S (2014) Combined aberrant expression of microRNA‐214 and UBC9 is an independent unfavorable prognostic factor for patients with gliomas. Med Oncol 31, 767. 24277415 10.1007/s12032-013-0767-5

[27] Liang T , Li L , Cheng Y , Ren C and Zhang G (2016) MicroRNA‐194 promotes the growth, migration, and invasion of ovarian carcinoma cells by targeting protein tyrosine phosphatase nonreceptor type 12. Onco Targets Ther 9, 4307–4315. 27486333 10.2147/OTT.S90976 PMC4956060

[28] Wang Z , Yin H , Zhang Y , Feng Y , Yan Z , Jiang X , Bukhari I , Iqbal F , Cooke HJ and Shi Q (2014) miR‐214‐mediated downregulation of RNF8 induces chromosomal instability in ovarian cancer cells. Cell Cycle 13, 3519–3528. 25483088 10.4161/15384101.2014.958413 PMC4615040

